# Wastewater-Based Surveillance of Human Adenoviruses in Italy: Quantification by Digital PCR and Molecular Typing via Nanopore Amplicon Sequencing

**DOI:** 10.3390/v17060791

**Published:** 2025-05-30

**Authors:** Carolina Veneri, G. Bonanno Ferraro, D. Congiu, A. Franco, D. Brandtner, P. Mancini, M. Iaconelli, The SARI Network, L. Lucentini, E. Suffredini, Giuseppina La Rosa

**Affiliations:** 1 National Center for Water Safety (CeNSia), Istituto Superiore di Sanità, 00161 Rome, Italy; carolina.veneri@iss.it (C.V.); giusy.bonannoferraro@iss.it (G.B.F.); daniele.congiu@iss.it (D.C.); agata.franco@iss.it (A.F.); pamela.mancini@iss.it (P.M.); marcello.iaconelli@iss.it (M.I.); luca.lucentini@iss.it (L.L.); 2 Department of Infectious Disease, Istituto Superiore di Sanità, 00161 Rome, Italy; david.brandtner@iss.it; 3 Department of Food Safety, Nutrition and Veterinary Public Health, Istituto Superiore di Sanità, 00161 Rome, Italy; elisabetta.suffredini@iss.it

**Keywords:** human adenoviruses (HAdV), wastewater-based epidemiology (WBE), digital PCR, nanopore sequencing

## Abstract

Wastewater-based epidemiology (WBE) offers valuable insight into viral circulation at the community level. In this study, we combined digital PCR (dPCR) with molecular typing to investigate the prevalence and diversity of human adenoviruses (HAdVs) in untreated wastewater samples collected throughout Italy. HAdV genomes were detected in over 93% of the 168 samples analyzed, with concentrations up to 4.5 × 10^6^ genome copies per liter. For genotypic characterization, we used nested PCR followed by Sanger and Oxford Nanopore Technologies (ONTs) long-read sequencing. While Sanger sequencing identified three dominant genotypes (HAdV-A12, HAdV-B3, and HAdV-F41), ONT sequencing provided enhanced resolution, confirming all previously identified types and revealing seven additional genotypes: HAdV-B21, HAdV-C5, HAdV-D45, HAdV-D46, HAdV-D49, HAdV-D83, and HAdV-F40. This comprehensive approach highlights the added value of ONT long-read sequencing in uncovering the genetic complexity of adenoviruses in wastewater, particularly in detecting rare or low abundance types that conventional methods may miss. Our findings highlight the value of integrating quantitative and high-resolution molecular tools in WBE to improve surveillance and better understand the epidemiology of viral pathogens circulating in the human population.

## 1. Introduction

Adenoviruses (AdVs) are non-enveloped viruses with icosahedral capsids and linear double-stranded DNA genomes, ranging from 25 to 48 kb. They are responsible for a broad spectrum of diseases across a wide range of animal species and are classified into six genera: *Mastadenovirus*, *Aviadenovirus*, *Atadenovirus*, *Siadenovirus*, *Ichtadenovirus*, and *Testadenovirus* [1]. Human adenoviruses (HAdVs), classified within the genus *Mastadenovirus*, comprise seven species (HAdV-A to HAdV-G), with as many as 116 types identified according to the Human Adenovirus Working Group (http://hadvwg.gmu.edu/, accessed on 1 April 2025).

Adenoviruses were first isolated from adenoid tissue in 1953 and are globally distributed pathogens capable of causing infections throughout the year. They exhibit considerable genetic diversity and are significant contributors to human disease, associated with a wide range of conditions, including mild respiratory infections, conjunctivitis, gastroenteritis, hepatitis, myocarditis, and potentially life-threatening pulmonary diseases. Although infections are generally mild and self-limiting in most individuals, they can pose serious health risks to vulnerable groups, including children, elderly, and immunocompromised individuals [2,3].

Adenovirus-related illnesses can appear in different epidemiological forms—as isolated cases, in endemic settings, or during outbreaks. Such outbreaks are commonly observed in schools, daycare centers, hospitals, summer camps, retirement homes, and military environments [4,5,6]. Transmission primarily occurs through close contact with infected individuals, as well as via the fecal–oral route or contaminated surfaces (fomites). Adenoviruses are shed in large quantities through bodily fluids during the acute phase of infection, including feces, respiratory secretions, and saliva. As a result, transmission routes are varied and include respiratory droplets, contaminated surfaces, and ingestion of virus-contaminated food or water. Adenoviruses are highly infectious, with an estimated average 50% infective dose (ID_50_) as low as 0.74 viral particles [7].

Adenoviruses are remarkably resilient to environmental stressors, including physical and chemical disinfectants. Their non-enveloped structure contributes to their stability, allowing them to persist on surfaces and in water for extended periods. Furthermore, adenoviruses are notably resistant to ultraviolet (UV) light, often requiring higher doses for effective inactivation compared to other viruses. They have been shown to persist in various aquatic environments, including drinking water, wastewater, and marine water, for several weeks [8,9,10]. Adenoviruses are frequently detected in different water sources, and waterborne outbreaks have been repeatedly reported [11,12].

A recent systematic review and meta-analysis confirmed the high prevalence of adenoviruses in wastewater worldwide, often exceeding 80% in untreated wastewater samples, and their frequent detection in other water sources, including recreational and drinking water [10]. While adenovirus detection in wastewater has been extensively documented, relatively few studies have specifically focused on identifying the circulating genotypes. This is particularly important because, in clinical practice, adenovirus infections—especially those causing gastroenteritis—are often not routinely tested for, and even when testing occurs, sequencing is rarely performed. As a result, we have limited knowledge of which specific adenovirus genotypes circulate within the population. This knowledge gap can be addressed through wastewater-based epidemiology (WBE), which provides a powerful tool for identifying and monitoring the adenovirus types circulating in the population. WBE has several advantages over traditional clinical surveillance, particularly in its ability to detect pathogens excreted by both symptomatic and asymptomatic individuals. This feature is particularly important for viruses such as adenoviruses, as infections are often undiagnosed due to mild or non-specific symptoms or because molecular typing is not routinely performed in clinical settings. By integrating signals from the entire contributing population, including those who do not seek medical care or undergo testing, WBE provides a more comprehensive and unbiased overview of circulating viral genotypes.

This study aims to provide a snapshot of the adenovirus genotypes circulating in the Italian population at the beginning of 2023, based on wastewater samples collected from multiple locations. While traditional molecular methods like PCR and Sanger sequencing are effective for detection, they are often limited in resolving mixed infections or identifying novel or rare types in complex environmental samples. In this regard, next-generation sequencing platforms, including second-generation methods like Illumina and third-generation technologies such as Oxford Nanopore Technologies (ONTs), offer significant advantages. Building upon previous work conducted by our team, in which we employed Illumina sequencing for next-generation sequencing (NGS) of adenovirus genotypes in wastewater [13], this study extends our approach by utilizing ONT for long-read sequencing which provide higher resolution and are expected to offer more accurate information about the circulating types of adenoviruses in wastewater. A secondary objective of this study is to quantify adenovirus in untreated wastewater samples. To achieve this, we employed digital PCR (dPCR), a technique that offers significant advantages over traditional real-time quantitative PCR (qPCR). Digital PCR provides absolute quantification of nucleic acids without the need for a standard curve, improving the accuracy of quantitative analysis. Moreover, being less susceptible to PCR inhibitors commonly found in complex environmental samples like wastewater, dPCR often displays higher sensitivity compared to real-time qPCR. This enhanced sensitivity makes dPCR particularly valuable for detecting less abundant viruses and genetic variants, even in samples with complex matrices. By combining genotypic identification with quantitative data, this study provides a structured approach to understanding adenovirus circulation and its potential public health impact.

## 2. Materials and Methods

### 2.1. Sampling, Viral Concentration, and RNA Extraction

A total of 168 wastewater samples were collected from 165 wastewater treatment plants (WWTPs) located in 18 regions and 2 autonomous provinces (APs) in Italy between 3 and 13 January 2023 (Table S1).

These samples were selected from those collected as part of the national SARS-CoV-2 surveillance program and submitted monthly to the Istituto Superiore di Sanità (ISS) for the so-called “flash surveys” on SARS-CoV-2 variants (https://www.iss.it/en/cov19-acque-reflue accessed on 1 April 2025). Sample collection, virus concentration, nucleic acid extraction, and quality assurance controls were carried out by the SARI network laboratories following a standardized national protocol, as previously described [14]. For virus concentration, sewage samples underwent heat-inactivation at 56 °C for 30 min, followed by polyethylene glycol (PEG)-based precipitation [15]. Briefly, 45 mL of each sample was subjected to centrifugation at 4500× *g* to remove solids. The supernatant (40 mL) was then transferred to fresh tubes, where 4 g of PEG 8000 and 0.9 g of NaCl were added. The mixture was continuously stirred at low temperature until complete PEG dissolution and subsequently centrifuged at 12,000× *g* for 2 h. The resulting pellet was resuspended in 2 mL of PBS for further processing.

Nucleic acid extraction was performed using commercially available magnetic silica-based extraction kits, followed by purification with the OneStep PCR Inhibitor Removal Kit (Zymo Research USA, Irvine, CA, USA). Quality control measures included the use of Mengovirus or Murine norovirus as process control viruses to assess recovery, as well as an inhibition control tested by real-time PCR to evaluate residual presence PCR inhibitors. Purified nucleic acids were subsequently shipped to the ISS and stored at −80 °C until further analysis.

Each sample, PCR assay, and primer/probe set was assigned a unique identifier in a dedicated laboratory database, accessible only to authorized users (https://biolabs.ddns.net/viramlab/ accessed on 1 April 2025) and used internally for tracking and quality assurance purposes.

### 2.2. Quantification of HAdV by dPCR Assay

All samples were analyzed by dPCR to quantify HAdV in wastewater, using a previously published qPCR assay targeting the hexon region (La Rosa et al., 2010 [16]), which was adapted for dPCR in this study. Primers and probes are shown in Table 1.

The dPCR experiments were performed on the QIAcuity One 5-plex dPCR system (Qiagen, Hilden, Germany), along with the QIAcuity UCP Probe PCR Kit (Qiagen, Hilden, Germany), using QIAcuity Nanoplate 8.5 k 96-well. Each reaction consisted of 12 μL of reaction mixture per well containing 3 μL of QIAcuity UCP Probe PCR MasterMix (4×), 0.4 μM of primer forward and reverse (ID 1080 and 1081) 0.2 μM of probe (ID 1082), and 4.2 µL of RNase-Free water. Finally, 3 μL of extracted sample nucleic acids were added as a template. For each sample, two technical replicates were performed. Nucleic acids were amplified under the following conditions: enzyme activation for 2 min at 95 °C and 40 cycles of 5 s at 95 °C and 30 s at 60 °C. Partitions were imaged with 300 ms (FAM) exposure time, with gain set to 6. Negative and positive controls were added in each run. Positive controls consisted of DNA extracted from reference strains of Human Adenovirus type 40 (ATCC^®^ VR-931™) and type 41 (ATCC^®^ VR-930™), obtained from the American Type Culture Collection (ATCC). The QIAcuity Software Suite version 3.1.0.0 was used to determine sample thresholds using positive and no-template control wells (NTCs) with the manual global threshold approach (Figure S1). PCR experiments were performed following the Minimum Information for Publication of Quantitative Digital PCR Experiments (MIQE) guidelines (https://pmc.ncbi.nlm.nih.gov/articles/PMC11625071/ accessed on 1 April 2025). The target concentration (copies/μL) in each sample was calculated using the instrument results and the formula:

HAdV (gc/L) = [dPCR result (copies/μL) × reaction volume/volume of tested DNA] × 100 × 25

where 100 is the total volume of extracted DNA and 25 is the ratio between the processed wastewater volume (40 mL) and the reference volume (1 L). Moreover, HAdV viral loads were normalized for both the daily flow rate of the corresponding WWTP and its population equivalents according to the following formula: genome copies (gc/L) × flow rate of WWTP in 24 h (L/day)/equivalent number of inhabitants served by the WWTP.

Quantitative data were categorized by geographical area (North, South, and Central Italy) and visualized as boxplots using Rstudio v2024.12.1 with ggplot v3.5.2 library.

### 2.3. Nested PCR Assays and Sanger Sequencing

A universal primer pair was used to amplify a fragment of the hexon gene targeting hypervariable regions HVR1 to 6, which successfully amplified all AdV species (Lu and Erdman, 2006 [17]). In the first PCR cycle (ID 582), primers AdhexF1 (ID 1551) and AdhexR1 (ID 1553) were used to amplify fragments ranging in length from 764 to 896 bps, depending on type and species. A second PCR (ID 583) was performed with the internal primers AdhexF2 (ID 1554) and AdhexR2 (ID 1555), yielding amplicons between 688 and 821 bp (Table 1). Amplification was performed using MyTaq™ Red Mix (Bioline Ltd., London, UK) in a 25 μL mixture containing 12.5 μL of the mix,1 μL of each primer (10 pmol), and 4 μL of the viral DNA. PCRs were run in a C1000 Touch PCR thermal cycler (Bio-Rad, Hercules, CA, USA) with the following thermal conditions: denaturation at 94 °C for 4 min, followed by 35 cycles at 94 °C for 1 min, at 45 °C for 1 min, and at 72 °C for 1 min, followed by a final 10 min extension cycle at 72 °C. After the first cycle of PCR amplification, 2 μL of the product was used for the nested PCR, under the same conditions. Standard precautions were implemented to avoid PCR contamination. After amplification, high-sensitivity capillary electrophoresis was performed using a QIAxcel instrument (Qiagen) and the QIAxcel DNA Fast Analysis Kit (50 bp–1.5 kb DNA Size Marker). PCR amplicons were purified using a Montage PCRm96 microwell filter plate (Millipore, Billerica, MA, USA). The purified PCR products were subjected to Sanger sequencing by BioFab Research (Rome, Italy), using the same nested PCR primers (ID 1554 and 1555). All sequences obtained in this study were submitted to GenBank under accession numbers from PV611558 to PV611626.

### 2.4. Next-Generation Sequencing

To overcome the limitations of Sanger sequencing in detecting less abundant sequence variants, long-read next-generation sequencing was performed using Oxford Nanopore technology. PCR-positive amplicons (2 μL for each) were pooled for sequencing.

Library preparation, sequencing, and bioinformatics analysis were performed as described in La Rosa et al. [14]. In brief, nanopore sequencing was conducted on a MinION device (Oxford Nanopore Technologies, Oxford, UK), using the cDNA-PCR Sequencing Kit (SQK-PCS109) along with native barcodes, as per the manufacturer’s instructions. A total of 25 fmol of the pooled library was loaded onto FLO-MIN106 (R9.4.1) flow cells, and sequencing was performed over a 72 h run using MinKNOW software (v4.5.4).

Basecalling was performed using the high-accuracy (HAC) model implemented in Guppy (v5.1.15), and demultiplexing was also conducted with Guppy on an Ubuntu 18.04 LTS workstation. Final FASTQ files were generated, and reads matching the expected PCR product lengths (688 and 821 bp) were selected. These reads were then aligned to a curated set of 111 reference adenovirus sequences (see Table S2) to exclude non-specific reads. A read was considered validly aligned to a reference if it met a minimum identity threshold of 97% and covered at least 95% of the query sequence. Additionally, a reference sequence was considered reliably detected only if the alignment depth reached a minimum of 30×.

### 2.5. Statistical and Bioinformatic Analyzes

**Figure 1:**
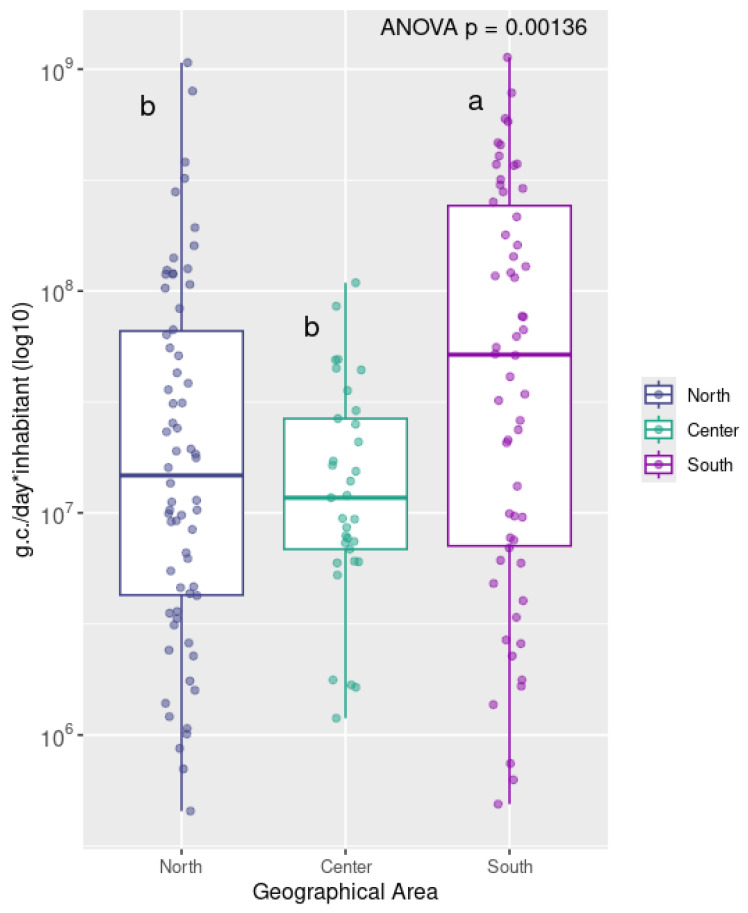
Normalized HAdV viral loads detected in Italian regions, grouped by geographical area (North, Center, South). Viral concentrations are expressed as genome copies per day per inhabitant and are log_10_-transformed. Groups North and Center are labeled with the letter “b” (not significantly different), while group South is labeled with the letter “a” (significantly different from both North and Center).

Statistical analyzes were performed using RStudio (v2024.12.1+402) base function. Multiple groups difference was tested with a one-way ANOVA and the specific pairwise differences between groups were tested with post hoc Tukey HDS tests. The ggplot2 package (v3.5.2) was used to generate boxplots in Figure 1, using log10-transformed values of normalized viral concentrations (genome copies/day/inhabitant), grouped by geographical macro-area (North, Center, South) and with the addition of text annotations to report statistical significance.

For phylogenetic analysis, nucleotide sequences were aligned with ClustalW as implemented in MEGA12 (Molecular Evolutionary Genetics Analysis, version 12). Model selection for phylogenetic inference was performed using the “Find Best DNA/Protein Models” function in MEGA12, which identified GTR as the best-fitting model. Tree robustness was evaluated using 1000 bootstrap replicates.

Nanopore reads were basecalled using Guppy (v5.1.15) with the high-accuracy (HAC) model and demultiplexed using Guppy barcoder. Quality filtering and read mapping were performed with minimap2 (v2.24) using default parameters. Reads were considered valid if they showed ≥97% identity and ≥95% query coverage against the reference set of 111 HAdV prototype sequences. Detection was confirmed only for genotypes with ≥30× coverage.

## 3. Results

The concentration and extraction procedures had an average recovery of 21.4% (range: 1–100%), with only one sample having a recovery below 1%. Regarding inhibition, all samples were within the acceptability criteria (ΔCq from reference reaction ≤ 2) with only one sample showing unacceptable inhibition (ΔCq > 2) (Table S1).

Human adenoviruses were detected in 157 out of 168 samples (93.5%) using dPCR, with viral concentrations ranging from 1.99 × 10^3^ to 4.46 × 10^6^ gc/L. Figure 1 shows the distribution of normalized human adenovirus (HAdV) concentrations detected in wastewater samples collected across North (regions of Aosta Valley, Piedmont, Liguria, Lombardy, Trentino-South Tyrol, Veneto, Friuli-Venezia Giulia, Emilia-Romagna), Center (regions of Tuscany, Umbria, Marche, Lazio), and South (regions of Abruzzo, Molise, Campania, Apulia, Basilicata, Calabria, Sicily) of Italy. Boxplots in Figure 1 were generated using log10-transformed values of normalized viral concentrations (genome copies/day/inhabitant), grouped by geographical macro-area.

**Figure 2:**
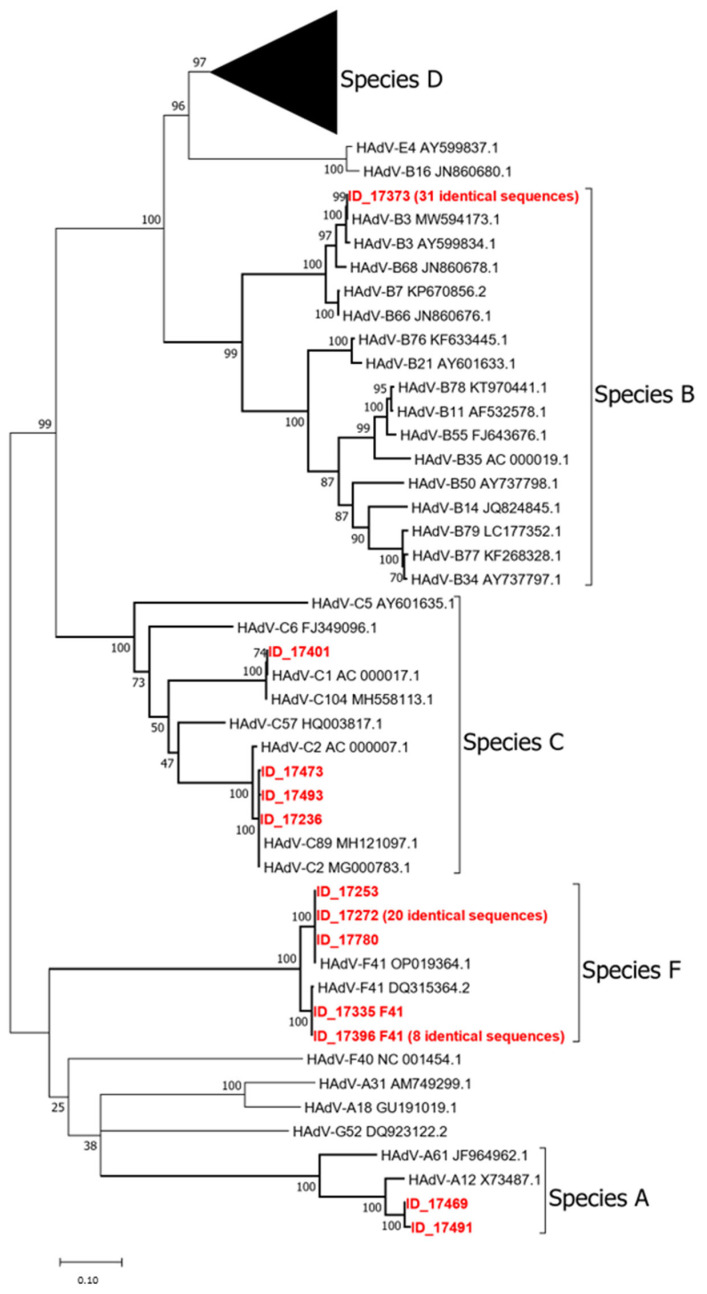
Phylogenetic tree of HAdVs sequences detected in this study: hexon region. The evolutionary history was inferred using the Maximum Likelihood method and General Time Reversible model. The tree is drawn to scale, with branch lengths measured in the number of substitutions per site. There were a total of 930 positions in the final dataset. Prototype strains belonging to species D were compressed into a triangle, as no study sequences from this species were present. Evolutionary analyzes were conducted in MEGA12.

**Figure 3:**
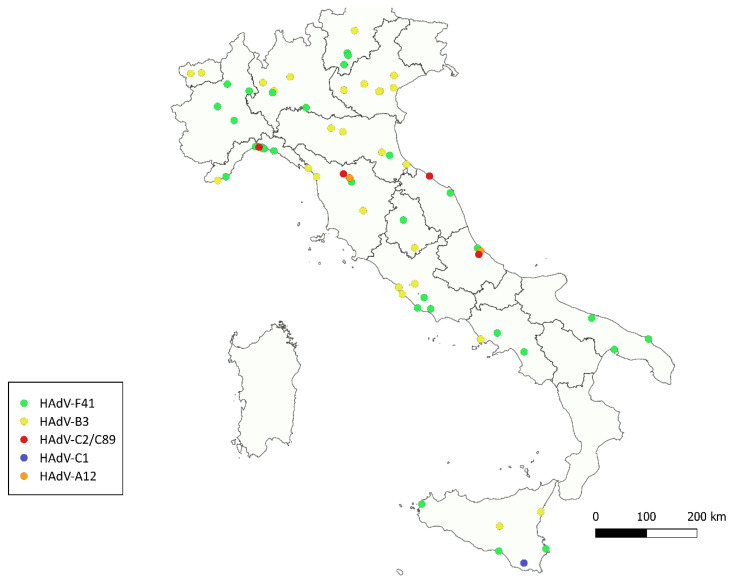
Geographic distribution of human adenovirus types identified in untreated wastewater samples collected across Italy. Each dot represents a sampling location, color-coded according to the detected adenovirus genotype: HAdV-F41 (green), HAdV-B3 (yellow), HAdV-C2/C89 (red), HAdV-C1 (blue), HAdV-A12 (orange).

Median HAdV concentrations were highest in the South, followed by Northern Italy and then by Central Italy. The South also exhibited the widest interquartile range (IQR), indicating greater variability among samples. Specifically, the South showed a median value around 10^8^ g.c./day*inhabitant, while the North and Center had median values closer to 10^7^. Detailed HAdV mean concentrations for each region are shown in Figure S2. The differences among the three geographical groups were statistically significant (*p*-value < 0.01) in a one-way ANOVA test. The following post hoc Tukey’s HSD test highlighted South group as the one significantly different from North and Center groups. Of the tested samples, 118 (70.2%) yielded successful amplification using the nested PCR assay. In 44 of these samples (37.3%), Sanger sequencing produced ambiguous or low-quality reads, likely due to mixed electropherogram (M.E.) signals associated with the presence of multiple viral variants, which are commonly found in environmental matrices. Conversely, a consensus sequence from high-quality forward and reverse reads was successfully obtained for 69 samples (58.5%), revealing the presence of four HAdV species: A, B, C, and F. Specifically, of these 69 samples, 66 were typed based on web BLAST (https://blast.ncbi.nlm.nih.gov/Blast.cgi accessed on 1 April 2025) analysis and phylogenetic analysis (see Figure 2): Species A was represented by HAdV-12 (2 samples); Species B by HAdV-3 (31 samples); Species C by HAdV-1 (1 samples); Species F by HAdV-41 (32 samples). In addition, four consensus sequences in Species C could not be unambiguously assigned to a single type as, in the analyzed region, they showed identical matches to two different prototypes. These were reported as type x/type y: HAdV-C2/89 (3 samples). An additional five samples could be partially characterized despite the lack of a consensus sequence, as only one of the forward or reverse reads was of high quality. Four of these were assigned to genotype F41 and one to C2/89. The geographical distribution of the different HAdV types is shown in Figure 3.

The analysis of the geographic distribution of human adenovirus genotypes in untreated wastewater samples revealed notable spatial variability. HAdV-F41 was the most widespread, detected across the entire country, including southern regions. HAdV-B3 was predominantly found in Northern Italy, with fewer occurrences in central and southern areas. HAdV-C2/89 was identified in a limited number of locations, mainly in Central Italy. HAdV-C1 appeared sporadically, with occurrences particularly in Sicily. Finally, HAdV-A12 was detected in a few areas of Central Italy.

The obtained amplicons showed the following lengths: 717 bp (type A12), 741/744 bp (type F41), 795 bp (type B3), 852 bp (type C1), and 855 bp (type C2/89).

Figure 3 shows the phylogenetic tree of the study sequences in comparison with 111 prototype strains (Table S2). Sequences for which a consensus could not be constructed (i.e., only the forward or reverse read was of good quality) were not included in the tree. To improve clarity, identical study sequences were collapsed, with only one representative included in the tree. The full list of identical samples is provided in Supplementary Table S3 for completeness. The phylogenetic analysis supported the classification of the detected HAdVs into species A, B, C, and F, with several sequences showing 100% nucleotide identity to their respective prototype strains. Additionally, several prototype strains belonging to species D, and occasionally some belonging to species C have identical sequences in the analyzed region, indicating that the assay is not sufficiently discriminatory for all HAdV types.

Long-read sequencing using Oxford Nanopore Technology (ONT) provided greater resolution, enabling the detection of a broader range of HAdV types. ONT sequencing confirmed all types identified by Sanger sequencing and additionally detected seven further types: HAdV-B21, HAdV-C5, HAdV-D45, HAdV-D46, HAdV-D49, HAdV-D83, and HAdV-F40, thereby highlighting the greater discriminatory power of this approach. The relative abundance (%) and number of reads of human adenovirus (HAdV) types detected by Sanger sequencing is illustrated in Table S4.

## 4. Discussion and Conclusions

Human adenoviruses circulate globally and can cause infections throughout the year, with no clear seasonal pattern. These infections are especially significant in vulnerable groups such as children under the age of two, elderly individuals, and immunocompromised adults [7]. Although human adenoviruses are recognized as common viral pathogens responsible for a wide range of infections, their epidemiological characterization remains largely incomplete, particularly regarding circulating genotypes. Indeed, adenovirus infections are often underdiagnosed in clinical settings, and when diagnosed, molecular typing is rarely performed. This lack of systematic clinical surveillance limits our understanding of genotype distribution and the temporal and geographical trends of HAdVs circulation. In this context, wastewater-based epidemiology (WBE) emerges as a valuable complementary tool, capable of capturing viral diversity at the population level, including asymptomatic and undiagnosed infections. By providing insight into the types co-circulating in the community, WBE can help fill critical knowledge gaps and support the early detection of emerging variants.

To contribute to a better understanding of HAdVs epidemiology, we conducted a nationwide study on untreated wastewater samples collected across Italy, with a dual objective: to quantify viral loads and to characterize circulating adenovirus genotypes. The samples were collected as part of the national SARS-CoV-2 wastewater surveillance program and specifically derived from the so-called flash surveys—rapid, monthly surveillance campaigns initiated in October 2021 and still ongoing.

HAdV genomes were detected in 93% of samples. This detection rate is higher than those reported in several previous studies, which typically relied on real-time PCR [18,19,20,21]. The increased sensitivity and robustness of digital PCR likely contributed to this higher detection frequency, especially in samples with low viral loads or high inhibitor content.

Our findings on viral concentrations (up to 4.5 × 10^6^ gc/L) are in line with earlier Italian studies, which reported HAdV concentrations ranging from 1.1 × 10^3^ to 6.2 × 10^8^ gc/L [16,22,23,24]. Globally, high concentrations of adenoviruses exceeding 10^6^ gc/L have been reported not only in Italy, but also in several other countries, including New Zealand [25], Egypt [21], the United States [26], Brazil [27,28], Uganda [29], and Australia [30]. This confirms that adenoviruses are commonly and abundantly excreted by the population, making them reliable indicators of human fecal contamination in wastewater.

Normalization of viral concentrations by flow rate and population equivalents enabled comparison across Italian macro-regions. The highest normalized loads were observed in the South, followed by the North and Center. The South also displayed the widest variability. The regional differences in HAdV concentrations observed in our study may reflect a combination of factors, including differences in virus circulation and in the structure and efficiency of wastewater networks. Variability in sewer coverage, retention times, and dilution effects may all influence the detected viral loads. Similar geographic variability in HAdV concentrations has been reported in other WBE studies and reviews, likely reflecting differences in epidemiology, infrastructure, and methodological approaches [10].

For adenovirus genotyping, a long fragment of the hexon gene was sequenced using both Sanger and next-generation sequencing (NGS) approaches. Among the samples successfully sequenced by Sanger, two genotypes clearly predominated: HAdV-F41 and HAdV-B3, together accounting for the majority of identified sequences. In addition to these predominant types, we also identified sequences belonging to species C. However, in several cases, the Sanger sequencing results could not unambiguously distinguish between closely related genotypes. Specifically, four samples matched equally with HAdV-C2 and HAdV-C89 and were accordingly reported as HAdV-C2/C89. This issue was further confirmed by our phylogenetic analysis, which included all currently recognized prototype sequences. The resulting tree showed that multiple prototype strains—especially within species D—shared identical sequences in the amplified region. This observation demonstrates that the hexon region originally considered suitable for discriminating among most HAdV genotypes (Lu and Erdman, 2006 [17]) is no longer sufficiently informative, as the number of classified types has grown. Consequently, relying solely on this target region can lead to ambiguities in genotyping, particularly when dealing with newly described or closely related types.

In addition to genotype diversity, we observed distinct geographical patterns in the distribution of adenovirus types. HAdV-F41, the most prevalent genotype, was widely distributed across all Italian macro-areas, including southern regions and the islands. HAdV-B3, the second most prevalent type, was predominantly found in Northern Italy, with limited occurrences elsewhere. Other genotypes, such as HAdV-C2, HAdV-C1, and HAdV-A12, were found in more localized regions, suggesting variable regional circulation dynamics. These spatial differences may reflect underlying demographic, infrastructural, or epidemiological factors influencing viral transmission and persistence in wastewater. No apparent spatial clustering was observed among the samples that produced mixed electropherogram signals and could not be reliably genotyped. Around 37% of the Sanger electropherograms were unreadable due to overlapping signals, likely reflecting co-infections with multiple types in the same sample. To overcome these limitations, we applied long-read sequencing using Oxford Nanopore Technology (ONT). The application of ONT provided deeper insights into the genotypic composition of circulating HAdVs. These findings are consistent with the previous literature suggesting that traditional sanger sequencing methods often underestimate the diversity of adenoviruses in complex matrices [13,31]. A qualitative concordance between Sanger and ONT results was observed: HAdV-F41 and HAdV-B3, the two most abundant types identified through ONT sequencing (Table S4), were also the most frequently detected by Sanger. It is important to note that Oxford Nanopore sequencing was performed on pooled PCR-positive amplicons, rather than on individual samples. As such, a direct one-to-one comparison between Sanger and ONT results for each specific sample was not feasible. This choice was dictated by practical constraints, including time, cost, and resource limitations. Future studies could benefit from applying ONT sequencing to individual amplicons, enabling a more precise genotypic characterization of adenoviruses, particularly in samples containing mixed infections or rare variants that may be overlooked with conventional approaches.

The genotypes identified in this study reflect the broad clinical spectrum and transmission routes associated with human adenoviruses. HAdV-F41, the most frequently detected genotype, is a well-known enteric adenovirus. In low-income countries, adenovirus types 40 and 41 are recognized as major contributors to diarrheal illness in children under two years old and are estimated to play a substantial role in the overall diarrheal disease burden among children under five [32,33]. Similarly, HAdV-F40, also detected in our pooled samples via ONT sequencing, belongs to the same enteric group and is frequently co-circulating with F41 in both clinical and environmental settings [34,35].

HAdV-B3, the second most common type in our study, is typically associated with respiratory diseases and is one of the most prevalent HAdV types detected in pharyngoconjunctivitis [36,37,38]. It has been implicated in several community outbreaks, especially in settings like schools [39,40]. HAdV-B3 has also been reported in many recreational waterborne outbreaks associated with swimming pools and spas [12]. The detection of this type in wastewater samples suggests active circulation in the population, including possible asymptomatic carriers.

Among the less frequent types detected only by NGS, several deserve attention due to their clinical associations. For example, HAdV-A12 is less common; however, it is known as the highly oncogenic types [41]. HAdV-A12 has been reported both in respiratory and gastrointestinal infections [42,43]. HAdV-B21, although rare, has been linked to severe respiratory disease, including pneumonia [44,45]. Types such as HAdV-D45, D46, D49, and D83 belong to species D, a highly diverse group often associated with ocular infections (e.g., epidemic keratoconjunctivitis) and, occasionally, with gastrointestinal or systemic disease, particularly in vulnerable hosts [46]. Finally, HAdV-C1 and HAdV-C5, members of species C (along with C2, C6, C57, C89, C104, and C108) are commonly found in early childhood infections and are known for their ability to establish persistent or latent infections, especially in lymphoid tissues [47,48].

A comparison between the adenovirus genotypes identified in wastewater and those reported in clinical respiratory infections in Italy during the same period further supports our findings. In a recent multicenter retrospective study conducted in Italy between 2022 and 2023 [37], genotyped clinical adenovirus-positive respiratory samples and identified six genotypes, with HAdV-B3 being the most prevalent (62%), followed by HAdV-C2 (19%), HAdV-C1 (12%), HAdV-C5 (4.4%), HAdV-B7 (1.9%), and HAdV-C6 (0.7%). Interestingly, the predominance of HAdV-B3 observed in clinical cases starting from early 2023 aligns with the detection of this genotype in wastewater samples collected during the same period. Moreover, the presence of HAdV-C types, particularly C1 and C2, among clinical isolates is consistent with their detection in environmental samples. This concordance suggests that wastewater surveillance can effectively mirror the trends observed in clinical settings, capturing both major and minor circulating genotypes within the population.

It is worth noting that, while clinical surveillance primarily identifies types associated with respiratory diseases, environmental monitoring also allows for the detection of a broader range of adenovirus types, including those linked to gastrointestinal infections, thereby offering a more comprehensive view of adenovirus circulation at the community level.

This study has several limitations. First, the sampling campaign consisted of a single nationwide collection conducted in January 2023. While this approach allowed for a snapshot of viral diversity across Italy, it does not account for seasonal fluctuations in HAdV circulation. Future studies should include repeated sampling over time to capture temporal trends and improve epidemiological interpretation. A second limitation concerns the use of pooled amplicons for long-read sequencing, which, although effective in revealing broader viral diversity, did not allow us to attribute specific genotypes to individual samples. Performing ONT sequencing at the single-sample level would improve the resolution of genotypic analyzes and help detect mixed infections with greater accuracy. While this approach was necessary due to practical constraints, it prevents a direct correlation between genotypic and quantitative data. Future work should aim to apply ONT sequencing at the individual-sample level to better link viral load with specific circulating types. Another limitation of our study is that molecular typing was based solely on the hexon gene. While this region is widely used for adenovirus classification, it does not allow for the identification of recombination events involving other genomic regions, such as the fiber or penton base genes. As a result, recombinant strains may go undetected or be misclassified. In clinical settings, multi-locus typing is recommended to ensure accurate genotyping. However, this approach is not easily applicable to complex environmental samples such as untreated wastewater, where viral genomes are fragmented and represent a composite signal from multiple excretors. Sequencing additional regions such as the fiber gene could nonetheless provide useful comparative information and help confirm genotyping results. Unfortunately, due to the limited availability of nucleic acid material—most of which was used for testing other viral targets including SARS-CoV-2 and influenza—amplification of additional regions was not feasible. In the future, expanding genotyping efforts to include additional genomic regions, such as the fiber gene, could improve the robustness of results by providing concordant information across loci and potentially revealing genotypes not detected through hexon-based typing alone. In summary, this study demonstrates the extensive presence and notable genetic diversity of HAdVs in untreated wastewater across Italy, highlighting the value of WBE for monitoring viral circulation at the population level. The use of Nanopore sequencing significantly improved genotypic resolution, enabling the detection of multiple co-circulating types within pooled samples, an aspect often missed by conventional methods like Sanger sequencing. However, it should be noted that the hexon gene region, while widely used, may not provide sufficient discriminatory power for closely related genotypes, particularly within species D, and in some cases, also within species C.

## Figures and Tables

**Table 1:** Primers and Probe used in this study.

Assay	PCR ID	Primer and Probe ID	Nucleotide Sequence (5′-3′)	Orientation	Amplicon Size (bp)	Reference
dPCR	494	1080	CWTACATGCACATCKCSGG	+	69	[16]
		1081	CRCGGGCRAAYTGCACCAG	-		
		1082	FAM-CCGGGCTCAGGTACTCCGAGGCGTCCT-TAMRA	-		
Nested PCR	582	1551	TICTTTGACATICGIGGIGTICTIGA	+	764 to 896	[17]
		1553	CTGTCIACIGCCTGRTTCCACA	-		
	583	1554	GGYCCYAGYTTYAARCCCTAYTC	+	688 to 821	
		1555	GGTTCTGTCICCCAGAGARTCIAGCA	-		

## Data Availability

The data presented in this study are available upon request from the corresponding author.

## Supplementary Materials

The following supporting information can be downloaded at: https://www.mdpi.com/article/10.3390/v17060791/s1, Figure S1: Examples of the scatterplot of a sample, positive and negative control; Figure S2: HAdV mean viral loads detected in Italian regions; Table S1: A total of 168 wastewater samples; Table S2: Prototype strains used in the phylogenetic analysis; Table S3: Full list of sample sequences omitted from phylogenetical analysis; Table S4: Relative abundance (%) and number of reads of human adenovirus (HAdV) types detected by Sanger sequencing.
