# Supplementary material for: Wastewater-Based Surveillance of Human Adenoviruses in Italy: Quantification by Digital PCR and Molecular Typing via Nanopore Amplicon Sequencing
Source: Viruses. 2025 May 30;17(6):791. doi: 10.3390/v17060791 (PMC12197328; doi:10.3390/v17060791)
Supplement: Supplementary file 1 [file viruses-17-00791-s001.zip › viruses-3653216-supplementary.pdf]

**Supplementary Materials:** Figure S1: Examples of the scatterplot of a sample, positive and negative control; Figure S2: HAdV mean viral loads detected in Italian regions; Table S1: A total of 168 wastewater samples; Table S2: Prototype strains used in the phylogenetic analysis; Table S3: Full list of sample sequences omitted from phylogenetical analysis; Table S4: Relative abundance (%) and number of reads of human adenovirus (HAdV) types detected by Sanger sequencing.

**Figure S1.** Examples of the scatterplot of a sample, positive and negative control

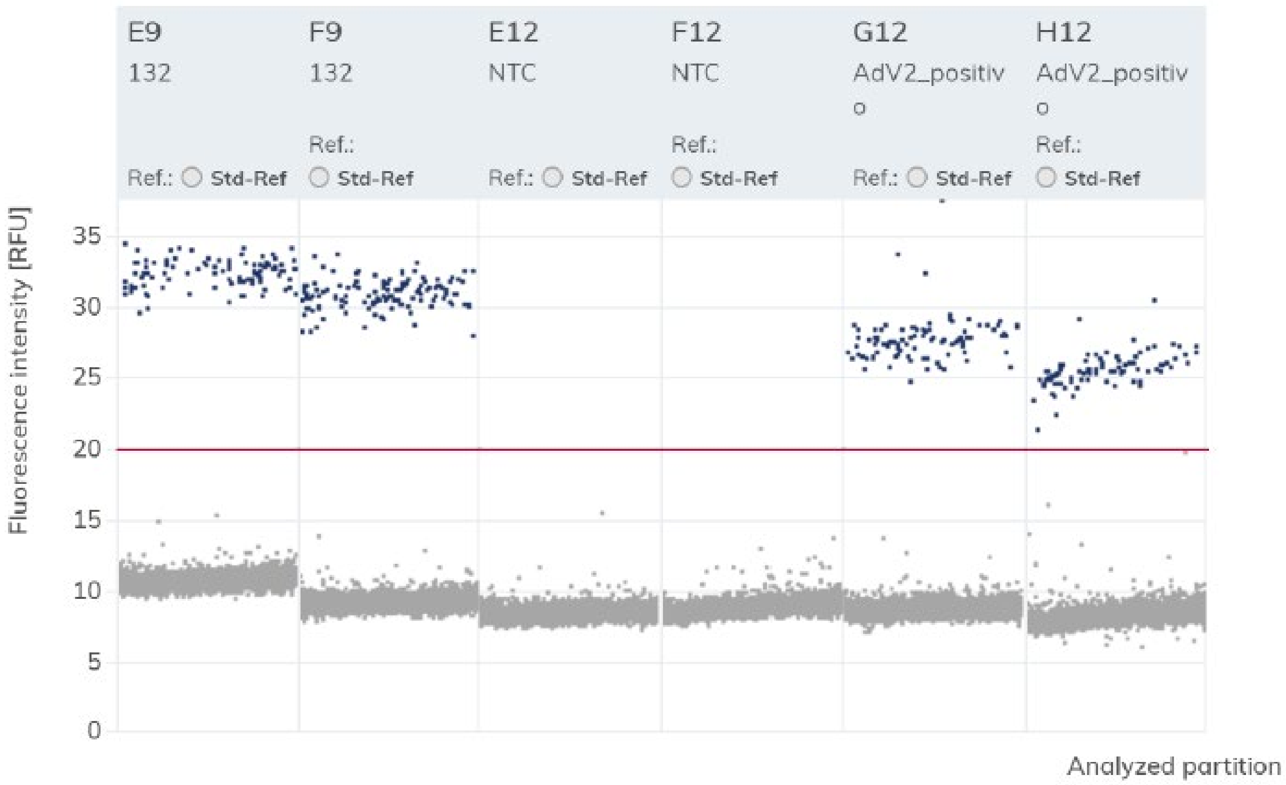

**Figure S2.** HAdV mean viral loads detected in Italian regions.

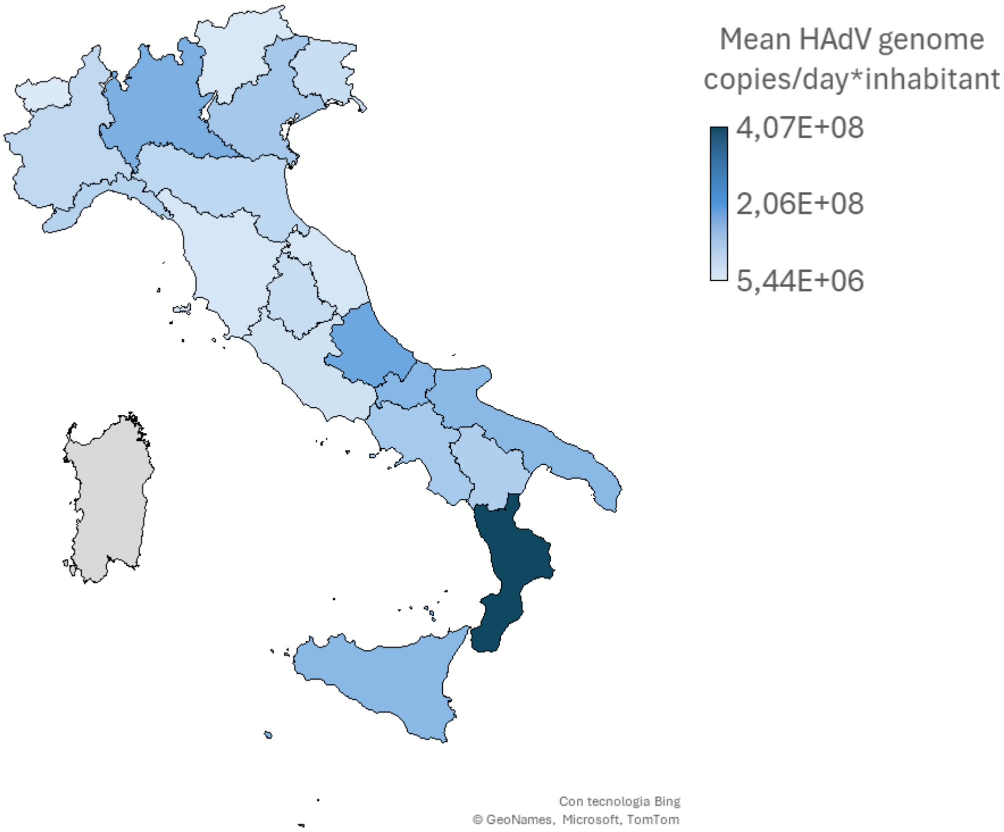


**Table S2.** Prototype strains used in the phylogenetic analysis

| N. | GenBank accession no. | Type | Reference |
|---|---|---|---|
| 1 | X73487.1 | HAdV-A12 | Ismail AM, et al. 2018 |
| 2 | GU191019.1 | HAdV-A18 | Ismail AM, et al. 2018 |
| 3 | AM749299.1 | HAdV-A31 | Ismail AM, et al. 2018 |
| 4 | JF964962.1 | HAdV-A61 | HAdV Working Group (March, 2024 update) |
| 5 | AY599834.1 | HAdV-B3 | Ismail AM, et al. 2018 |
| 6 | KP670856.2 | HAdV-B7 | Ismail AM, et al. 2018 |
| 7 | AF532578.1 | HAdV-B11 | Ismail AM, et al. 2018 |
| 8 | JQ824845.1 | HAdV-B14 | Ismail AM, et al. 2018 |
| 9 | JN860680.1 | HAdV-B16 | Ismail AM, et al. 2018 |
| 10 | AY601633.1 | HAdV-B21 | Ismail AM, et al. 2018 |
| 11 | AY737797.1 | HAdV-B34 | Ismail AM, et al. 2018 |
| 12 | AC000019.1 | HAdV-B35 | Ismail AM, et al. 2018 |
| 13 | AY737798.1 | HAdV-B50 | Ismail AM, et al. 2018 |
| 14 | FJ643676.1 | HAdV-B55 | HAdV Working Group (March, 2024 update) |
| 15 | JN860676.1 | HAdV-B66 | HAdV Working Group (March, 2024 update) |
| 16 | JN860678.1 | HAdV-B68 | HAdV Working Group (March, 2024 update) |
| 17 | KF633445.1 | HAdV-B76 | HAdV Working Group (March, 2024 update) |
| 18 | KF268328.1 | HAdV-B77 | HAdV Working Group (March, 2024 update) |

| | | | |
|---|---|---|---|
| 19 | KT970441.1 | HAdV-B78 | HAdV Working Group (March, 2024 update) |
| 20 | LC177352.1 | HAdV-B79 | HAdV Working Group (March, 2024 update) |
| 21 | ON393912.1 | HAdV-B106 | HAdV Working Group (March, 2024 update) |
| 22 | OR853835.1 | HAdV-B114 | HAdV Working Group (March, 2024 update) |
| 23 | AC000017.1 | HAdV-C1 | Ismail AM, et al. 2018 |
| 24 | AC000007.1 | HAdV-C2 | Ismail AM, et al. 2018 |
| 25 | AY601635.1 | HAdV-C5 | Ismail AM, et al. 2018 |
| 26 | FJ349096.1 | HAdV-C6 | Ismail AM, et al. 2018 |
| 27 | HQ003817.1 | HAdV-C57 | HAdV Working Group (March, 2024 update) |
| 28 | MH121097.1 | HAdV-C89 | HAdV Working Group (March, 2024 update) |
| 29 | MH558113.1 | HAdV-C104 | HAdV Working Group (March, 2024 update) |
| 30 | AB448767.1 | HAdV-D8 | Ismail AM, et al. 2018 |
| 31 | AJ854486.1 | HAdV-D9 | Ismail AM, et al. 2018 |
| 32 | JN226746.1 | HAdV-D10 | Ismail AM, et al. 2018 |
| 33 | JN226747.1 | HAdV-D13 | Ismail AM, et al. 2018 |
| 34 | KF268204.1 | HAdV-D15 | Ismail AM, et al. 2018 |
| 35 | HQ910407.1 | HAdV-D17 | Ismail AM, et al. 2018 |
| 36 | JQ326209.1 | HAdV-D19 | Ismail AM, et al. 2018 |
| 37 | JN226749.1 | HAdV-D20 | Ismail AM, et al. 2018 |
| 38 | FJ619037.1 | HAdV-D22 | Ismail AM, et al. 2018 |
| 39 | JN226750.1 | HAdV-D23 | Ismail AM, et al. 2018 |
| 40 | JN226751.1 | HAdV-D24 | Ismail AM, et al. 2018 |
| 41 | JN226752.1 | HAdV-D25 | Ismail AM, et al. 2018 |
| 42 | EF153474.1 | HAdV-D26 | Ismail AM, et al. 2018 |
| 43 | JN226753.1 | HAdV-D27 | Ismail AM, et al. 2018 |
| 44 | FJ824826.1 | HAdV-D28 | Ismail AM, et al. 2018 |
| 45 | JN226754.1 | HAdV-D29 | Ismail AM, et al. 2018 |
| 46 | JN226755.1 | HAdV-D30 | Ismail AM, et al. 2018 |
| 47 | JN226756.1 | HAdV-D32 | Ismail AM, et al. 2018 |
| 48 | JN226758.1 | HAdV-D33 | Ismail AM, et al. 2018 |
| 49 | GQ384080.1 | HAdV-D36 | Ismail AM, et al. 2018 |
| 50 | AB448775.1 | HAdV-D37 | Ismail AM, et al. 2018 |
| 51 | JN226759.1 | HAdV-D38 | Ismail AM, et al. 2018 |
| 52 | JN226760.1 | HAdV-D39 | Ismail AM, et al. 2018 |
| 53 | JN226761.1 | HAdV-D42 | Ismail AM, et al. 2018 |
| 54 | JN226762.1 | HAdV-D43 | Ismail AM, et al. 2018 |
| 55 | JN226763.1 | HAdV-D44 | Ismail AM, et al. 2018 |
| 56 | JN226764.1 | HAdV-D45 | Ismail AM, et al. 2018 |
| 57 | AY875648.1 | HAdV-D46 | Ismail AM, et al. 2018 |
| 58 | JN226757.1 | HAdV-D47 | Ismail AM, et al. 2018 |
| 59 | EF153473.1 | HAdV-D48 | Ismail AM, et al. 2018 |
| 60 | DQ393829.1 | HAdV-D49 | Ismail AM, et al. 2018 |
| 61 | JN226765.1 | HAdV-D51 | Ismail AM, et al. 2018 |
| 62 | FJ169625.1 | HAdV-D53 | HAdV Working Group (March, 2024 update) |
| 63 | AB333801.2 | HAdV-D54 | HAdV Working Group (March, 2024 update) |
| 64 | HM770721.2 | HAdV-D56 | HAdV Working Group (March, 2024 update) |

| | | | |
|---|---|---|---|
| 65 | HQ883276.1 | HAdV-D58 | HAdV Working Group (March, 2024 update) |
| 66 | JF799911.1 | HAdV-D59 | HAdV Working Group (March, 2024 update) |
| 67 | HQ007053.1 | HAdV-D60 | HAdV Working Group (March, 2024 update) |
| 68 | JN162671.1 | HAdV-D62 | HAdV Working Group (March, 2024 update) |
| 69 | JN935766.1 | HAdV-D63 | HAdV Working Group (March, 2024 update) |
| 70 | EF121005.1 | HAdV-D64 | HAdV Working Group (March, 2024 update) |
| 71 | AP012285.1 | HAdV-D65 | HAdV Working Group (March, 2024 update) |
| 72 | AP012302.1 | HAdV-D67 | HAdV Working Group (March, 2024 update) |
| 73 | JN226748.1 | HAdV-D69 | HAdV Working Group (March, 2024 update) |
| 74 | KP641339.1 | HAdV-D70 | HAdV Working Group (March, 2024 update) |
| 75 | KF268207.1 | HAdV-D71 | HAdV Working Group (March, 2024 update) |
| 76 | KF268335.1 | HAdV-D72 | HAdV Working Group (March, 2024 update) |
| 77 | KY618676.1 | HAdV-D73 | HAdV Working Group (March, 2024 update) |
| 78 | KY618677.1 | HAdV-D74 | HAdV Working Group (March, 2024 update) |
| 79 | KY618678.1 | HAdV-D75 | HAdV Working Group (March, 2024 update) |
| 80 | KY618679.1 | HAdV-D80 | HAdV Working Group (March, 2024 update) |
| 81 | AB765926.1 | HAdV-D81 | HAdV Working Group (March, 2024 update) |
| 82 | LC066535.1 | HAdV-D82 | HAdV Working Group (March, 2024 update) |
| 83 | KX827426.1 | HAdV-D83 | HAdV Working Group (March, 2024 update) |
| 84 | MF416150.1 | HAdV-D84 | HAdV Working Group (March, 2024 update) |
| 85 | LC314153.1 | HAdV-D85 | HAdV Working Group (March, 2024 update) |
| 86 | KX868297.2 | HAdV-D86 | HAdV Working Group (March, 2024 update) |
| 87 | MF476841.1 | HAdV-D87 | HAdV Working Group (March, 2024 update) |
| 88 | MF476842.1 | HAdV-D88 | HAdV Working Group (March, 2024 update) |
| 89 | KF268208.1 | HAdV-D91 | HAdV Working Group (March, 2024 update) |
| 90 | KF268325.1 | HAdV-D92 | HAdV Working Group (March, 2024 update) |
| 91 | KF268334.1 | HAdV-D93 | HAdV Working Group (March, 2024 update) |
| 92 | KF268201.1 | HAdV-D94 | HAdV Working Group (March, 2024 update) |
| 93 | KF268206.1 | HAdV-D95 | HAdV Working Group (March, 2024 update) |
| 94 | KF268327.1 | HAdV-D96 | HAdV Working Group (March, 2024 update) |
| 95 | KF268320.1 | HAdV-D97 | HAdV Working Group (March, 2024 update) |
| 96 | KF268332.1 | HAdV-D98 | HAdV Working Group (March, 2024 update) |
| 97 | KF268211.1 | HAdV-D99 | HAdV Working Group (March, 2024 update) |
| 98 | KF268330.1 | HAdV-D100 | HAdV Working Group (March, 2024 update) |
| 99 | KF268324.1 | HAdV-D101 | HAdV Working Group (March, 2024 update) |
| 100 | KF268312.1 | HAdV-D102 | HAdV Working Group (March, 2024 update) |
| 101 | KF268322.1 | HAdV-D103 | HAdV Working Group (March, 2024 update) |
| 102 | ON393913.1 | HAdV-D105 | HAdV Working Group (March, 2024 update) |
| 103 | MK174992.1 | HAdV-D107 | HAdV Working Group (March, 2024 update) |
| 104 | LC652931.1 | HAdV-D111 | HAdV Working Group (March, 2024 update) |
| 105 | MW694832.1 | HAdV-D113 | HAdV Working Group (March, 2024 update) |
| 106 | OR044915.1 | HAdV-D115 | HAdV Working Group (March, 2024 update) |
| 107 | AY599837.1 | HAdV-E4 | Ismail AM, et al. 2018 |
| 108 | NC001454.1 | HAdV-F40 | Ismail AM, et al. 2018 |
| 109 | DQ315364.2 | HAdV-F41 | Ismail AM, et al. 2018 |
| 110 | OP019364.1 | HAdV-F41 | GenBank |

| 111 | DQ923122.2 | HAdV-G52 | GenBank |
|---|---|---|---|

**Table S3.** Full list of sample sequences omitted from phylogenetical analysis

| Specie | Reference prototype (A.N.) | Reference sample (ID) | Redundant (identical) sample sequence (ID) |
|---|---|---|---|
| HAdV-B3 | MW594173.1 | 17373 | 17778,17425,17426,17427, 17429, 17438, 17911, 17914, 17397, 17347, 17405,17516, 17411,17442,17403,17459,17460,17474,17372, 17420, 17421, 17292, 17293, 17325, 17326, 17327, 17363, 17364, 17365, 17915 |
| HAdV-F41 | DQ315364 | 17335, 17396 | 17355, 17278, 17259, 17406, 17432, 17304, 17256, 17240 |
| HAdV-F41 | OP019364.1 | 17780, 17253, 17272 | 17492, 17331, 17333, 17387,17388,17390, 17392, 17398, 17374, 17302, 17258, 17354, 17254, 17301, 17328, 17443, 17404, 17467, 17774 |

**Table S4.** Relative abundance (%) and number of reads of human adenovirus (HAdV) types detected by Sanger sequencing.

| HAdV reference | nr of reads | rel. ab. (%) |
|---|---|---|
| HAdVB3_AY599834.1 | 139095 | 47,09 |
| HAdVC89_MH121097.1 | 68024 | 23,03 |
| HAdVF41_DQ315364.2 | 31792 | 10,76 |
| HAdVC2_AC_000007.1 | 19782 | 6,70 |
| HAdVC104__MH558113.1 | 16852 | 5,71 |
| HAdVC1_AC_000017.1 | 7259 | 2,46 |
| HAdVD73_KY618676.1 | 4154 | 1,41 |
| HAdVC5_AY601635.1 | 2085 | 0,71 |
| HAdVA12_X73487.1 | 1432 | 0,48 |
| HAdVD46_AY875648.1 | 1201 | 0,41 |
| HAdVD81_AB765926.1 | 1139 | 0,39 |
| HAdVD83_KX827426.1 | 888 | 0,30 |
| HAdVB66_JN860676.1 | 642 | 0,22 |
| HAdVB21_AY601633.1 | 294 | 0,10 |
| HAdVD49_DQ393829.1 | 188 | 0,06 |
| HAdVD23_JN226750.1 | 177 | 0,06 |
| HAdVF40_NC_001454.1 | 126 | 0,04 |
| HAdVD45_JN226764.1 | 50 | 0,02 |
| HAdVD82_LC066535.1 | 48 | 0,02 |
| HAdVD56_HM770721.2 | 38 | 0,01 |
| HAdVD111_LC652931.1 | 36 | 0,01 |
| HAdVD9_AJ854486.1 | 33 | 0,01 |
| HAdVD95_KF268206.1 | 31 | 0,01 |
